# Fatty acid patterns of dog erythrocyte membranes after feeding of a fish-oil based DHA-rich supplement with a base diet low in n-3 fatty acids versus a diet containing added n-3 fatty acids

**DOI:** 10.1186/1751-0147-53-57

**Published:** 2011-10-24

**Authors:** Katja Stoeckel, Leif Højvang Nielsen, Herbert Fuhrmann, Lisa Bachmann

**Affiliations:** 1 Institute of Physiological Chemistry, An den Tierkliniken 1, D - 04103 Leipzig, Germany; 2 Boehringer Ingelheim Denmark A/S, Natural Care Center Strødamvej 52, DK-2100, København, Denmark; 3 Institute of Animal Nutrition, Nutrition Diseases and Dietetics, Gustav-Kühn-Straße 8 D - 04159 Leipzig, Germany

**Keywords:** eicosapentaenoic acid, dogs, PUFA, dietary manipulation

## Abstract

**Background:**

In dogs, increasing the tissue n-3 fatty acid (FA) content is associated with potential benefit in some medical conditions, e.g. atopic dermatitis, cancer or heart disease. Therefore effectively and conveniently increasing tissue n-3 FA levels in dogs is of interest. Incorporation of dietary n-3 FA into cell membranes may be studied by FA analysis of erythrocyte membranes (EM), because of the correlation of its FA composition with the FA composition of other cells. Aim of the study was to determine whether an n-3 FA additive added to a control diet is as effective in increasing EM n-3 FA content as feeding an n-3 FA enriched diet. Furthermore the time course of the incorporation of dietary n-3 FA into canine EM was investigated.

**Methods:**

Thirty dogs were randomly divided into three dietary groups with ten dogs per group. CONT got a dry dog food diet which did not contain EPA or DHA. FO got a dry dog food diet with a high EPA and DHA content. ADD got the CONT diet combined with an n-3 FA additive rich in DHA and EPA. After a feeding period of 12 weeks the additive was discontinued in ADD and these dogs were fed CONT diet for another four weeks to observe washout effects. Erythrocyte lipids were extracted from venous blood samples and their FA composition was determined by gas chromatography. The Mann-Whitney-U-test was used to detect significant differences between the different groups and time points.

**Results:**

After one week the proportions of n-3 FA, DHA and EPA were already significantly increased in ADD and FO, apparently reaching a plateau within eight weeks. In our study DHA and not EPA was preferably incorporated into the EM. After discontinuing the administration of the additive in ADD, the n-3 FA values declined slowly without reaching baseline levels within four weeks.

**Conclusions:**

In dogs, an increase of dietary n-3 FA content leads to a rapid inclusion of n-3 FA into EM, regardless of whether the n-3 FA are offered as an enriched diet or as a normal diet supplemented with an n-3 FA additive.

## Background

Polyunsaturated fatty acids (PUFA) such as arachidonic acid (AA), docosahexaenoic acid (DHA), eicosapentaenoic acid (EPA), and linoleic acid (LA), which are present in cell membrane lipids, are important for many biochemical and physiological functions[1]. The proportion of the different types of fatty acids (FA) in the cell membrane influences the properties of the membranes[2] and the release of inflammatory mediators[3]. The mechanism likely to be responsible for the potential benefits of n-3 FA in inflammatory diseases is that the eicosanoids produced from the breakdown of n-3 FA are less potent inflammatory mediators than those produced from n-6 FA[3]. Therefore, increasing the dietary level of n-3 PUFA has the potential to prevent or treat a variety of medical conditions in humans and animals, e.g. arthritis, some cancers, cardiovascular disease, depression or neurological diseases[4]. Also in dogs, the consumption of n-3 PUFA has been linked to beneficial effects on atopic dermatitis[5], cancer[6], heart disease[7] and chronic renal failure[8].

Among the n-3 FA EPA and DHA are generally considered to be the FA that have the most beneficial effects. Controversy exists about which n-3 fatty acid is responsible for the main effect of n-3 FA supplementation. Although dual supplementation with both EPA and DHA was found to have beneficial effects, there have been some studies in rodents suggesting that for a certain disease one n-3 FA might be more beneficial than the other. One study comparing the anti-inflammatory effects of DHA and EPA in induced contact dermatitis in mice determined that although both EPA and DHA can ameliorate dermatitis, EPA appears to be more potent[9]. In another study with an induced mammary tumor model in rats, DHA was found to be more effective than EPA in suppressing mammary carcinogenesis[10]. In human medicine, the inclusion of plentiful DHA in the diet is considered to improve learning ability and the development of the brain[11]. If these findings are verified in dogs, the ability to selectively increase the incorporation of either DHA or EPA into cell membranes according to the therapeutic goal, would be desirable as part of the disease management.

Fish and fish oils are rich in long chain n-3 FA, mostly EPA and DHA[12], whereas terrestrial plants only provide alpha linolenic acid (ALA) [13]. Although ALA can be converted to EPA, which can then be elongated to DHA, in dogs the conversion of ALA to EPA is less efficacious than providing dietary EPA. For DHA this effect is even more pronounced than for EPA, since the conversion rate is even lower[14]. Therefore increasing tissue EPA and especially DHA levels via dietary manipulation would be of interest for the diseased dog. Since a complete change of the diet is not always possible in dogs requiring a certain diet due to health problems or might not be acceptable to the dog owner, we sought to determine if supplementing a diet that is low in n-3 FA with an n-3 FA additive is as effective in increasing membrane n-3 FA content and especially membrane EPA and DHA content. When using dietary intervention in a clinical setting it would also be of interest to know when and to which extent changes in the fatty acid composition of the cell membranes can be expected.

The erythrocyte membrane (EM) is one of the prototypes of cell membrane that has been extensively studied as a model because of its ready availability[2]. Since FA composition of the EM correlates with that of other cell membranes, the effect of dietary FA supplementation may be studied by FA analyses of EM[1,15]. In previous studies, significant correlations have been found between PUFA intake and incorporation of PUFA into erythrocyte membranes[16]. Thus, monitoring incorporation of n-3 FA into EM is a suitable model for monitoring the FA intake[17].

The aim of the study was to determine whether an n-3 FA additive added to a control diet not containing any EPA or DHA, is as effective in increasing membrane n-3 FA content as a complete change of the diet to an n-3 FA enriched diet. Furthermore we sought to investigate the time course of the incorporation of dietary n-3 FA into canine EM using an n-3 FA supplemented diet and an n-3 FA enriched diet in comparison with a control diet.

## Materials and methods

### Animals

Thirty clinically healthy, intact Beagle dogs (fifteen male, fifteen female; mean age of 2.2 ± 0.65 years) were chosen for the present study. The dogs were pair-housed in kennels and fed individually. Individual weights were taken at the beginning of the study and the amounts of dog food were rationed according to the recommendations of the manufacturer of the diets (g food per kg body weight). Water was provided *ad libitum *all day. The study was carried out at Summit Ridge Farms, Susquehanna, PA, USA. Experiments were approved by the Institutional Animal Care and Use Committee and conducted in accordance to the principles and specific guidelines presented in the *Guide for the Care and Use of Laboratory Animals *published by the US National Institute of Health.

For eight weeks up to the onset of the study the dogs were fed with a dry dog food diet (Joy Special Meal, Joy Pet Foods, St. Marys, Ohio, USA) that does not contain any PUFA from fish oil. This diet served as a control diet during the 12 week study period. The dogs were fed once a day at 8 am. Individual food intake was determined daily by weighing the individual feeding bowls before and after feeding.

### Experimental Design

The study was a prospective open label, randomised study. Prior to the onset of the study, the included dogs were randomly assigned to three groups with ten dogs per group (five males; five females). Dogs in the control group (CONT) were fed 300 g of the control diet, dogs in the FA diet group (FO) were fed 250 g of a commercial dog food (Eukanuba^® ^Medium Breed Puppy) containing fish oil (FO) and dogs in the additive (ADD) group were fed 300 g of the control diet supplemented by syringe with 0.09 ml (= 85 mg) per kg of body weight of a fish oil concentrate (Refortin^®^, according to the manufacturers' recommendations). All diets were given for 12 weeks. After 12 week study period the ADD group got the control diet without the additive for another 4 weeks to observe washout effects. The n-3 FA additive used had an energy content of 38.0 KJ/g and contained 14% EPA and 48% DHA. The analytical composition and FA patterns of the diets are listed in Table 1. Analysis of the diets was done by Eurofins Steins laboratory A/S, Vejen, Denmark.

**Table 1:** Analytical composition of the dog food products and fatty acid (FA) pattern (%) of dog food.

Components	Joy special meal	Eukanuba^® ^medium breed puppy
Raw fat	11·0%	18·0%
Crude protein	27·0%	29·0%
Crude fiber	4·0%	4·0%
Moisture	10·0%	8·0%
energy content	14·6 kJ/g	18·0 kJ/g

Fatty acid pattern		

	FA in g/kg total diet	
	
LA*^1^	19·69	35·64
AA†^1^	0·25	1·13
ALA‡^2^	2·86	1·76
SDA**|**	0·2	0·16
EPA^2^	-	1·66
DHA^2^	-	1·1
Total n-3	3·08	5·22
Total n-6	20·35	37·8
Total n-7 FA	5·09	14·33
Total n9 FA	39·8	64·49
Total SFA	38·61	54·0
Ratio total n-6/total n-3	6·61/1	7·24/1

Data are based on an as fed basis. The FA included for the calculation of total n-3, total n-6 and total saturated FA are listed in additional file 1.

*LA, linoleic acid; †AA, arachidonic acid; ‡ALA, α-linolenic acid; **| **SDA, stearidonic acid; ^1 ^n-6 FA ^2 ^n-3 FA

Blood samples were taken by jugular vein puncture six hours after feeding at week 0, 1, 2, 4, 8 and 12, and additionally at week 14 and 16 for ADD. EDTA was used as anticoagulant. After sampling syringes with blood were immediately put on ice. The health of all dogs was monitored by routine clinical examinations.

### Measurements and analyses

Immediately after blood sampling erythrocyte ghosts were prepared as described previously[18,19]. Blood samples were centrifuged at 4°C for 5 minutes at 865 g and the plasma was aspirated. Erythrocytes were washed three times in a 5-fold volume of cold saline (pH = 7.4) with subsequent centrifugation at 4°C for 5 minutes at 2862 g. Leukocytes and saline were aspirated after each washing. The remaining erythrocyte pellet was lysed in 5 mM phosphate buffer (volume ratio: 1:7). The lysed erythrocytes were kept frozen at -80°C until lyophilisation. The dried pellets were weighed and kept frozen until further use (-80°C).

Total erythrocyte lipids were extracted with hexane and transmethylated with methanolic HCl containing 0.2% butylhydroxytoluol as antioxidant[20]. FA concentrations were analysed by measuring fatty acid methyl esters via gas chromatography with di-C17-phosphatidylcholine as internal standard, as previously described[18,21]. Methyl esters were measured with a Varian CP 3800 gas chromatograph (Varian, Darmstadt, Germany) equipped with a flame ionization detector. The chromatogram was analysed by means of the program Star 5.5 (Varian), with the internal standard as reference peak. In total 44 FA were determined. For the calculation of total n-3 and total n-6 FA in the diet the sum of all n-3 and n-6 FA analysed by Eurofins Steins laboratory A/S, Vejen, Denmark, with a chain length between 18 and 22 carbon atoms was calculated, namely C 18:3n3 (ALA), C 18:4n3, C 20:4n3, C 20:5n3 (EPA), C 22:5n3 (docosapentaenoic acid, DPA) and C 22:6n3 (DHA) for calculation of total n-3 FA and C 18:2n6 (LA), C 18:3n6, C 20:2n6, C 20:4n6 (AA), C 22:4n6 and C 22:5n6 for calculation of total n-6 FA. In EM, we were additionally able to detect small amounts of the n-3 FA C 20:3n3 and C 22:4n3 and of the n-6 FA C 20:3n6 and C 22:2n6, which were also used for the calculation of the total amount of n-3 and n-6 FA of EM, respectively. The fatty acids that were included in the calculated amount of total n-7, n-9 and saturated fatty acids in the diets and in EM are listed in additional file 1. The FA pattern calculated on a molar base is given in %. Areas under the curves (AUC) of the percentage of EPA and DHA in EM over the 12 weeks of the study period were calculated by the software SigmaPlot (version 11, Systat Software, Erkrath, Germany) in order to determine the response to FA administration over time.

### Statistical Analyses

Since the Levene test for homogeneity of variances showed significant differences within the study period for some FA, we decided to use nonparametric tests for all FA and groups. Hence, data were expressed as median (± quartile). For the FA of interest the Kruskal-Wallis test was performed. For those that showed statistically significant differences over time the Mann-Whitney-U-Test was computed for comparison of the individual time points within the group. The same test was used for comparison between groups at the different time points. Statistical analysis was conducted with PASW (version 18, SPSS Inc., Chicago, IL, US) and a two-sided p-value ≤ 0.01 was considered to be statistically significant.

## Results

### Animals

The experimental feeding was well tolerated by all dogs as appetite was maintained and no clinical signs were found in routine clinical examinations. The compliance to the diet was high throughout the study. As the body weights of the dogs were similar (Table 2) all dogs within the same group got the same amount of food and the dose of the additive was also similar among the dogs of ADD (0.92 ml ± 0.17 ml) during the study period. As the amount of food in ADD was not corrected for the energy content supplied by the additive and the manufacturers' recommendations were slightly different between CONT and FO, energy intake in dogs in ADD and FO was slightly higher than that from dogs in CONT (0.77% and 2.74%, respectively). However, all dogs maintained their body weight during the study (± 1.2 kg). The average daily FA consumption per kg body weight in the three test groups is given in Table 2.

**Table 2:** Age and weight of the dogs (mean ± SD) at baseline and at week 12 of experimental feeding as well as daily fatty acid (FA) intake per kg body weight in control (CONT), additive (ADD) and fish oil (FO) group.

	CONT		ADD		FO	
	
	Mean	SD	Mean	SD	Mean	SD
Age (years)	2.2	1	2.1	0.3	2.3	0.5

Weight (kg) week 1	10.9	0.69	10.3	0.65	10.7	0.83

week 12	11.2	0.72	10.5	0.64	11.4	0.84

FA intake						

	mg/d/kg body weight					
	
LA*^1^	483.3		516.1		742.5	
AA†^1^	6.2		6.6		23.6	
ALA‡^2^	70.2		75		36.8	
SDA**|**	4.8		5.1		3.6	
EPA^2^	-		11.9		34.5	
DHA^2^	-		40.8		22.9	
Total n-3	75.6		142.2		108.8	
Total n-6	499.5		533.4		787.5	

The FA included for the calculation of total n-3 and total n-6 FA are listed in additional file 1.

*LA, linoleic acid; †AA, arachidonic acid; ‡ALA, α-linolenic acid; **| **SDA, stearidonic acid; ^1 ^n-6 FA ^2 ^n-3 FA

### Fatty acid composition of erythrocyte membranes

The FA baseline levels at week 0 did not differ significantly among the dietary groups (all p ≥ 0.02). There were no significant differences in the percentage of n-7, n-9 and saturated FA between the groups at any time point except for week 12 when the content of total n-7 FA was significantly higher in FO than in ADD (p ≤ 0.01). Likewise, there were very few statistically significant differences in the proportion of n-7, n-9 and saturated FA in CONT, ADD and FO between the different time points within the respective group (all p ≤ 0.01) (additional file 2).

**Figure 1:**
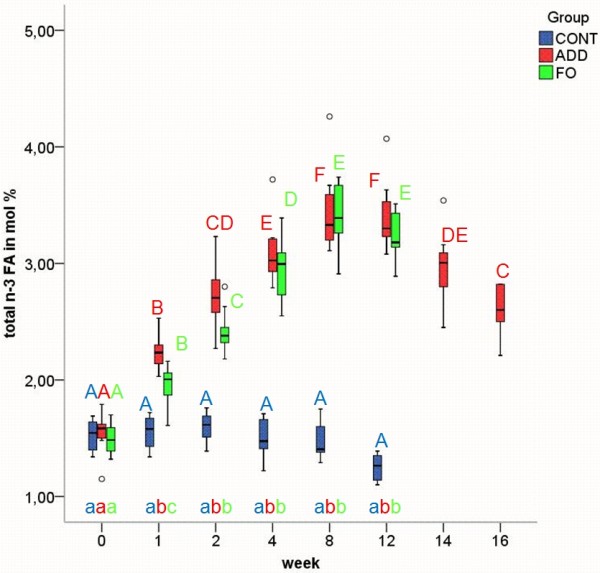
**n-3 fatty acids (FA) in erythrocyte membranes in the three different dietary groups**. Median and quartiles of the percentage of n-3 FA in erythrocyte membranes in control (CONT), additive (ADD) and fish oil (FO) group at week 0, 1, 2, 4, 8, 12, 14 and 16 of experimental feeding. Different uppercase letters indicate statistically significant differences between different time points within the individual groups. Different Lowercase letters indicate statistically significant differences between groups at the same time point. (p ≤ 0.01). The FA included for the calculation of total n-3 FA are listed in additional file 1.

**Figure 2:**
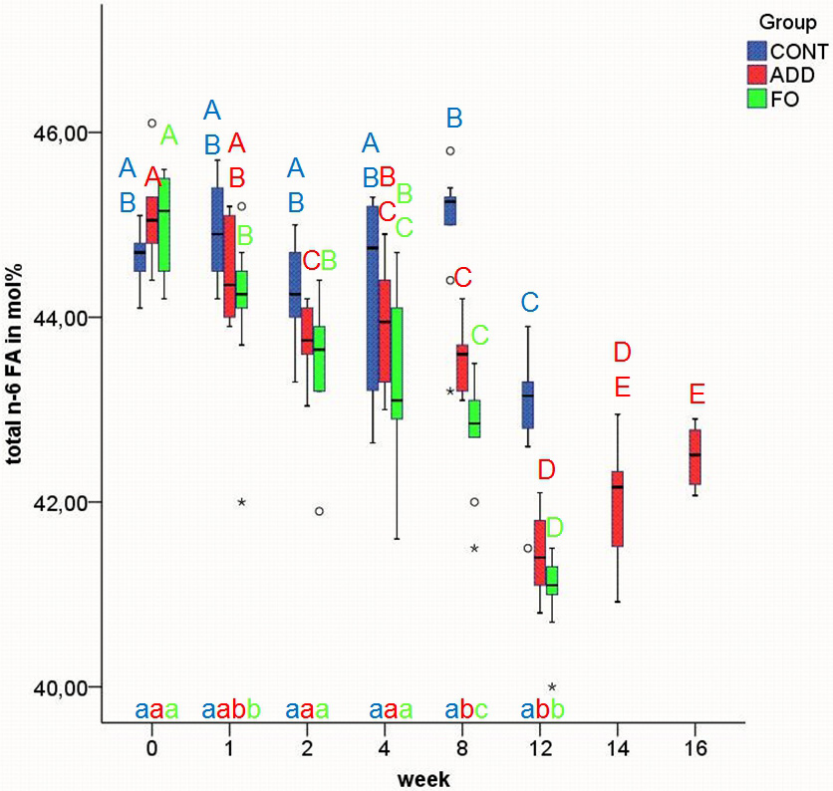
**n-6 fatty acids (FA) in erythrocyte membranes in the three different dietary groups**. Median and quartiles of the percentage of n-6 FA in erythrocyte membranes in control (CONT), additive (ADD) and fish oil (FO) group at week 0, 1, 2, 4, 8, 12, 14 and 16 of experimental feeding. Different uppercase letters indicate statistically significant differences between different time points within the individual groups. Different Lowercase letters indicate statistically significant differences between groups at the same time point. (p ≤ 0.01). The FA included for the calculation of total n-6 FA are listed in additional file 1.

Erythrocyte n-3 FA content at baseline was 1.5% of total FA (Figure 1). Both n-3 diets significantly increased the amount of n-3 FA in EM after the first week (p ≤ 0.01), apparently reaching a plateau within eight weeks (Figure 1). The rise in n-3 FA (mainly EPA and DHA) in ADD and FO during the feeding period was compensated by a decrease in n-6 FA as shown in Figure 2. In FO significant reductions in n-6 FA content in comparison with the control group could be observed at week 2, 8 and 12 (all p ≤ 0.01). In ADD the amount of n-6 FA did not decrease significantly in comparison with the control group until week 8 (p ≤ 0.01). In CONT, n-6 FA were significantly lower at week 12 compared to all other time points and showed significant differences between week 2 and 8 (all p ≤ 0.01). Due to the changes in n-6 and n-3 FA the n-6/n-3-ratio changed from 30/1 (week 1) to 12, 5/1 (week 12) in both ADD and FO.

**Figure 3:**
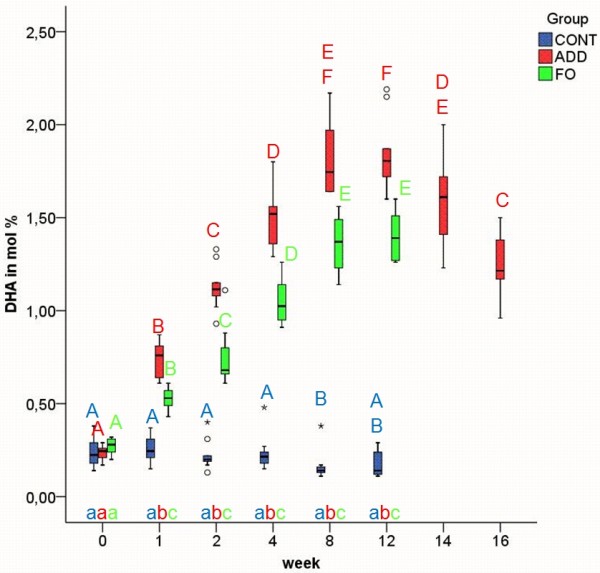
**Docosahexaenoic acid (DHA) in erythrocyte membranes in the three different dietary groups**. Median and quartiles of the percentage of DHA in erythrocyte membranes in control (CONT), additive (ADD) and fish oil (FO) group at week 0, 1, 2, 4, 8, 12, 14 and 16 of experimental feeding. Different uppercase letters indicate statistically significant differences between different time points within the individual groups. Different Lowercase letters indicate statistically significant differences between groups at the same time point. (p ≤ 0.01).

**Figure 4:**
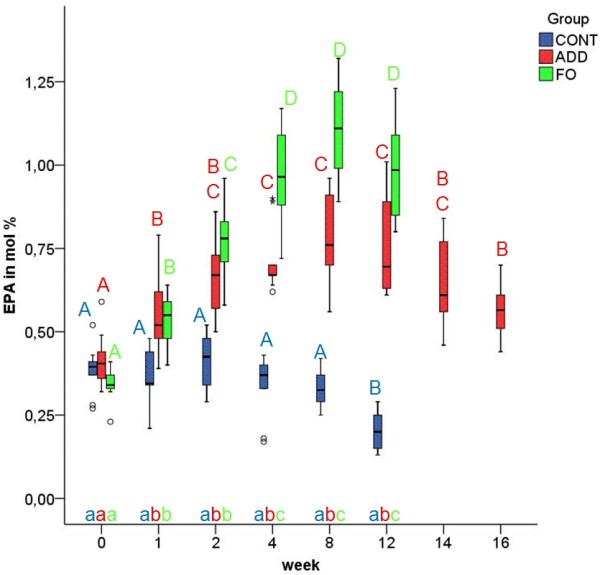
**Eicosapentaenoic acid (EPA) in erythrocyte membranes in the three different dietary groups**. Median and quartiles of the percentage of EPA in erythrocyte membranes in control (CONT), additive (ADD) and fish oil (FO) group at week 0, 1, 2, 4, 8, 12, 14 and 16 of experimental feeding. Different uppercase letters indicate statistically significant differences between different time points within the individual groups. Different Lowercase letters indicate statistically significant differences between groups at the same time point. (p ≤ 0.01).

In ADD, the relative amounts of EPA (Figure 3) and DHA (Figure 4) in EM increased gradually and significantly during supplementation (p ≤ 0.01) and did not return to their baseline levels within the 4 weeks of the washout period. During the whole study period the values of EPA and DHA in EM of ADD and FO differed significantly from those before supplementation and from those of CONT (p ≤ 0.01). In ADD, the amount of EPA in EM reached a plateau within two weeks already, whereas in FO, it took 4 weeks for EPA to be equilibrated (Figure 3). According to the different content of DHA and EPA in ADD and FO the increase of DHA was more pronounced in ADD (Figure 4), whereas EPA reached higher values in FO (Figure 3). The percentage of DHA in EM showed statistically significant differences between the n-3 dietary groups throughout the period of feeding (week 1 until week 12) (p ≤ 0.01). The amount of EPA significantly differed at week 4, 8 and 12 between ADD and FO (p ≤ 0.01). In CONT EPA showed statistically significant differences at week 12 compared to all other time points (p ≤ 0.01). DHA in CONT showed significant differences between the first 4 weeks of the study and week 8, whereas there were no significant differences between week 12 and all other time points (all p ≥ 0.02).

After an initial increase at week 1 DPA showed a steady decrease in ADD until week 12. However, statistically significant differences could only be observed between week 1 and all time points from week 8 until the end of the study and between week 2 and 12 (additional file 3) (p ≤ 0.01). There were no statistically significant differences in DPA membrane content in CONT and FO during the study period (p ≥ 0.77 and p ≥ 0.18, respectively).

In FO ALA decreased significantly from week 1 (p ≤ 0.01) apparently reaching a plateau at week 8. In ADD and CONT, there were no significant changes in ALA content at any point in time (p ≥ 0.94 and p ≥ 0.42, respectively).

**Figure 5:**
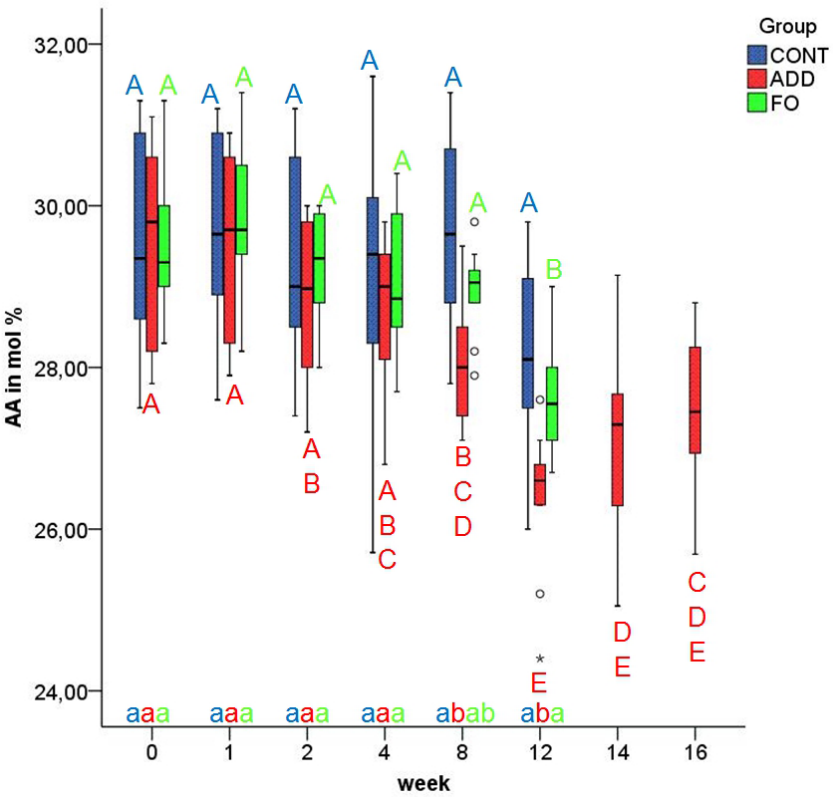
**Arachidonic acid (AA) in erythrocyte membranes in the three different dietary groups**. Median and quartiles of the percentage of AA in erythrocyte membranes in control (CONT), additive (ADD) and fish oil (FO) group at week 0, 1, 2, 4, 8, 12, 14 and 16 of experimental feeding. Different uppercase letters indicate statistically significant differences between different time points within the individual groups. Different Lowercase letters indicate statistically significant differences between groups at the same time point. (p ≤ 0.01).

No changes were observed in the percentage of AA in EM of CONT during the whole study. In ADD and FO, the median AA proportions (Figure 5) decreased gradually during supplementation from 29.5%, to the lowest concentration (26.6%) at week 12 in ADD. Significant differences between initial values and AA content in EM of ADD after supplementation were observed from week 8 until the end of the study (p ≤ 0.01). At week 12 the proportion of erythrocyte AA in ADD differed significantly from those of CONT and FO (p ≤ 0.01).

The membrane content of LA did not change significantly in any group during the study (all p ≥ 0.01).

**Figure 6:**
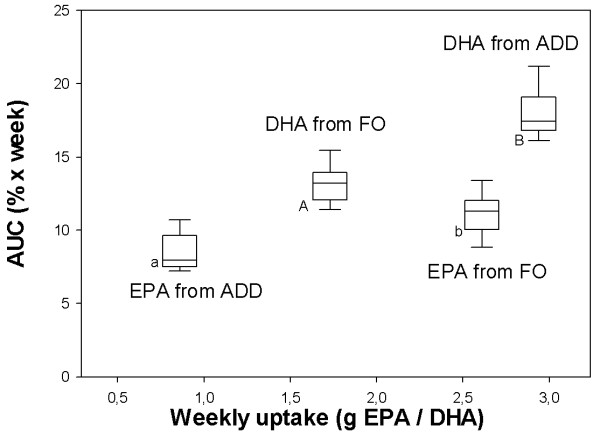
**Area under the curve (AUC) of EPA and DHA in EM in ADD and FO in relation to fatty acid intake**. Median, Quartiles and range of area under the curve (AUC) of EPA and DHA in erythrocyte membranes of FO and ADD in relation to fatty acid (FA) intake of EPA or DHA in g per week. Uppercase letters indicate statistically significant differences between ADD and FO in AUC of DHA. Lowercase letters indicate statistically significant differences between ADD and FO in AUC of EPA. (p ≤ 0.01).

The AUC of DHA and EPA in EM (Figure 6) increased according to the dietary intake of DHA and EPA. However, the AUC of DHA (ADD: 17.46 (16.83; 19.08) FO: 13.21 (12.08; 13.95)) was higher than that of EPA (ADD: 7.97 (7.52; 9.63) FO: 11.33 (10.07; 12.05)).

After changing to CONT feeding in ADD (week 12), the altered FA values declined slowly or increased, respectively, without reaching baseline levels within the four weeks of the washout period (Figures 1, 2, 3, 4, 5).

## Discussion

We compared the incorporation of n-3 FA into EM in dogs fed three types of diet; a control diet containing low amounts of EPA and DHA, the control diet supplemented with an n-3 FA additive and a commercial dog food containing n-3 FA from fish oil. The initial FA proportions of the EM are consistent with previous observations except for total n-6 FA and AA. These values (44.5 and 29.5%, respectively) were higher than in a previous study in rats[16] and much higher than in erythrocytes of human subjects consuming no additional n-3 FA with their habitual diet[22]. This might be due to different AA and n-6 FA content in the diets or to species specific differences in the FA composition of cell membranes [19]. In ADD and FO, DHA and EPA in EM were already elevated after one week. In several studies it was observed that diets containing different amounts of n-3 FA led to increased values of these PUFA in EM[12,22-25]. Our data suggest that this increase can be achieved with an enriched diet as well as with a normal diet supplemented with an n-3 FA additive. To our knowledge no study exists observing such a rapid change in erythrocyte FA of dogs as in our study. These results agree with the data of Kew et al.[26] in mice. In their study, feeding high levels of EPA or DHA resulted in significantly increased splenocyte EPA or DHA after one week of supplementation. Our results are also consistent with the findings of Hansen et al.[27], in which canine serum EPA and DHA concentrations were significantly increased after one week of fish oil supplementation. In contrast, other researchers found significant differences to initial values not before week 2 or later. However, many of these studies were conducted with other species and started the first sampling at week 2 or later [12,22-25].

FA could be incorporated into EM during erythropoiesis, or by interaction of mature erythrocytes with plasma FA[28]. In dogs the average lifespan of erythrocytes is 17 weeks[29]. Hence, the significant increase of FA in erythrocytes during the first week of administration of n-3 PUFA we observed in our study cannot be explained by entry of new erythrocytes into the circulation[25], but indicates a rapid exchange between erythrocyte and plasma FA. The exchange of FA between plasma and blood cells may occur by different routes, e.g. the transfer of albumin-bound nonesterified FA through FA binding proteins or transfer of phospholipids from plasma lipoproteins. Some authors suggest that there is an active protein-mediated transport of FA into EM[30]. Furthermore, evidence exists that exchange of FA in EM occurs as a lipid-mediated process via flip-flop and to a lesser extent by dissociation[31]. Our results suggest that FA transfer from plasma to erythrocytes plays a predominant role in determining EM FA composition when dietary fat intake is changed. This was also shown by Orton et al.[17]. We cannot exclude the possibility that after prolonged periods of intervention, a sustained increase in n-3 FA of erythrocytes may occur during replacement of erythrocytes. Nevertheless, it is improbable for erythrocyte turnover to make a major contribution to the early changes in n-3 FA content of the EM observed in our study.

The increase of DHA in EM as calculated by its AUC reached higher values in ADD than in FO (Figure 6). The proportions of DHA and EPA in ADD are different from those of FO. Consequently, we assume that DHA-rich supplements have a greater ability to advance DHA content in cell membranes than other fish oil based PUFA diets rich in EPA. If future *in-vivo *studies confirm beneficial effects of DHA supplementation in dogs, this fact may be advantageous in dietary support of disorders or conditions in dogs, like cancer or in the nutrition of the developing puppy, where DHA is found to be more beneficial than EPA.

In the present study, DHA and not EPA was preferably incorporated into the EM. This fact agrees with the study of Milte et al.[24], in which the increase of human erythrocyte DHA was more pronounced than that of EPA after consumption of a DHA-rich fish oil. Our results conflict with the study of Brown et al.[12] in which human erythrocyte EPA was strongly correlated to the amount of EPA in the diet, while the change in erythrocyte DHA was correlated with the duration of the feeding period irrespective of the amount of DHA. A preferential incorporation of EPA was also observed in humans receiving a EPA-rich fish oil diet[32]. Our data also contradict the results of Jude et al.[33] in dogs. In this study a fish oil-enriched diet containing twice as much DHA as EPA showed a preferential incorporation of EPA. These conflicting results might be due to previous dietary exposure to FA[28] or to different FA sources, formulations, proportions and concentrations.

In accordance with the increase of n-3 FA, n-6 FA were significantly lowered in ADD and FO compared to baseline levels, but these values did not show significant differences to the n-6 FA in CONT until week 8. The incorporation of n-3 FA at the expense of AA has been shown earlier by Jude et al.[33] and Kew et al.[26]. There was also a significant reduction of n-6 FA in FO, despite the fact, that the daily uptake of n-6 FA in this group (787.5 mg/d/kg body weight) was higher than in ADD (533.4 mg/d/kg body weight). Although the content of AA in FO is about three times higher than in CONT and ADD (Table 2), an increase of erythrocyte AA in FO was not observed. It is possible that the EM already reached their maximum AA content during the 8 weeks before the onset of the study, when all animals got the CONT diet. This would mean that, even though the FO diet has a higher AA content than the CONT diet, no further increase of EM AA content could be expected. Furthermore, the high EPA content in the FO diet possibly reduced AA incorporation in EM. Despite FO and CONT having similar dietary ratios of n-6 to n-3 FA (Table 1) and the dietary n-6 to n-3 FA ratio of ADD being much smaller than that of CONT or FO (3.75:1 vs 6.61:1 and 7.24:1) the ratio of n-6 to n-3 FA content in EM at the end of the study did not differ between ADD and FO. In a study examining the influence of dietary n-3 FA on canine plasma FA composition Hall et al. could show that up to certain dietary concentrations the dose of n-3 FA administered, independent of the n-6 to n-3 FA ratio, determined the plasma n-3 FA composition[34]. Our results suggest that the same is true for EM. However, in another study it was shown that alterations of canine plasma FA composition appeared to depend on both the dose of dietary n-3 FA and the n-6 to n-3 ratio[35]. Therefore, it might be possible that combining dietary n-3 PUFA with low levels of AA can be even more effective in diminishing cellular AA than increasing dietary n-3 FA alone. By diminishing cellular AA the release of inflammatory mediators can be reduced and the health status of patients with chronic inflammatory diseases could be improved[3,36]. Our data suggest a steady state of incorporation and release of EPA and DHA in EM. These data are comparable with earlier results of plasma and neutrophil FA in dogs [37], supporting the concept of a competitive and "saturable" hyperbolic relationship between dietary n-3 PUFA, plasma and tissue FA in dogs. The results of the present study are to some extent consistent with the findings that the equilibration of erythrocyte FA is dependent on abundance and proportion of the different FA in the diet. However, our data cannot exclude that there are other factors such as previous dietary exposure to n-6 FA and source or formulation of the dietary lipids that might influence the equilibration process of FA in cellular membranes. Since in contrast to n-3 FA, n-6 FA were not equilibrated within the 12 weeks of the observation time it is important to conduct further studies in order to determine the equilibration time of n-6 FA. A slow equilibration process of n-6 FA could also explain the fact that, in spite of the rapid response of EM n-3 FA content, it takes a few weeks to observe beneficial effects of dietary n-3 FA supplementation in dogs[36,38].

The period after finishing n-3 FA supplementation in ADD in the intent to observe re-establishment of baseline levels was too short. Nevertheless, the increased erythrocyte n-3 FA content significantly declined and n-6 FA content significantly increased during this period. These data indicate that for studies dealing with dietary n-3 FA in dogs a washout period of > 4 weeks is required. According to Brown et al.[12], for EPA it takes 18 weeks to return to baseline levels in dogs, whereas DHA has an extended washout period (> 18 weeks).

Although there are many studies on the incorporation of FA into cell membranes, the factors which influence FA inclusion, except for FA concentration in test meals, remain unclear. More research on the time course of incorporation of PUFA into EM and other cell membranes is needed to clarify whether it depends on the concentration, the formulation of these lipids or if there are species specific effects.

## Conclusions

Moderate n-3 PUFA intake in dogs leads to incorporation of DHA and EPA in erythrocytes in a dose-dependent manner. Significant changes of membrane total n-3 FA, EPA and DHA were observable from the first week of the study. The content of these FA in EM reached a plateau no later than eight weeks. In contrast, total n-6 FA and AA were not equilibrated after 12 weeks, suggesting that the decrease of n-6 FA membrane content in reaction to dietary changes might be delayed. In our study, the additive combined with a dry dog food diet containing no EPA or DHA was as effective in increasing total n-3 FA as changing the diet to a dry dog food diet with a high EPA and DHA content. These findings indicate that it might be possible to supplement an existing diet with an n-3 fatty acid additive instead of completely changing the diet. For dogs with dietary intolerance, this is a substantial improvement. Further studies are needed to determine whether increasing dietary n-3 FA is as effective and leads to the same rapid increase of EM n-3 FA content in diseased dogs as in the healthy dogs used in our study.

## Competing interests

Research was supported by Boehringer Ingelheim Denmark A/S, København. KS received a student fellowship financed by Boehringer Ingelheim Denmark; LN is employed by Boehringer Ingelheim Denmark; HF, GD and LB have no conflicts of interest. The Freundeskreis Tiermedizin e.V. of the Faculty of Veterinary medicine of the University of Leipzig covered the article-processing charge.

## Authors' contributions

LN and HF designed and directed the research. GD conducted the laboratory analyses. KS, LB and HF analysed the data, wrote the paper and had primary responsibility for final content. All authors read and approved the final manuscript.

## Supplementary Material

Additional file 1 **List of fatty acids included for calculation of total n-3, total n-6, total n-7, total n-9 and total saturated FA in diet and EM**. Click here for file

Additional file 2 **FA analysed in EM for CONT, ADD and FO (median and quartiles)**. Click here for file

Additional file 3 **Median 2^nd ^and 3^rd ^quartiles of the proportions of linoleic acid (LA), alpha linolenic acid (ALA) and docosapentaenoic acid (DPA) in erythrocyte membranes in control (CONT), additive (ADD) and fish oil (FO) group before and at week 1, 2, 4, 8, 12, 14 and 16 of experimental feeding**. Click here for file
