# Supplementary material for: Fatty acid patterns of dog erythrocyte membranes after feeding of a fish-oil based DHA-rich supplement with a base diet low in n-3 fatty acids versus a diet containing added n-3 fatty acids
Source: Acta Vet Scand. 2011 Oct 24;53(1):57. doi: 10.1186/1751-0147-53-57 (PMC3213045; doi:10.1186/1751-0147-53-57)
Supplement: Additional file 1 — List of fatty acids included for calculation of total n-3, total n-6, total n-7, total n-9 and total saturated FA in diet and EM. [file 1751-0147-53-57-S1.PDF]

List of fatty acids included for calculation of total n-3, total n-6, total n-7, total n-9 and total saturated FA in diet and EM

FA included for calculation of total n-3, total n-6, total n-7, total n-9 and total saturated FA in EM. FA discussed in the paper are marked in bold script and names are given in the column behind where appropriate.

**n-3 FA**		**n-6 FA**		n-7 FA	n-9 FA	saturated FA
C 18:3n3	LNA	C 18:2n6		C 16:1n7	C 16:1n9	C 14:0
C 18:4n3		C 18:3n6	LA	C 17:1n7	C 18:1n9	C 15:0
C 20:3n3		C 20:2n6		C 18:1n7	C 18:2n9	C 16:0
C 20:4n3		C 20:3n6		C 18:2n7	C 20:1n9	C 18:0
**C 20:5n3**	**EPA**	**C 20:4n6**	**AA**	C 20:1n7	C 20:2n9	C 20:0
C 22:4n3		C 22:2n6		C 20:2n7	C 20:3n9	C 22:0
C 22:5n3	DPA	C 22:4n6		C 20:3n7	C 22:1n9	C 23:0
**C 22:6n3**	**DHA**	C 22:5n6		C 22:1n7	C 22:2n9	C 24:0
					C 22:3n9	
					C 24:1n9	

FA included for calculation of total n-3, total n-6, total n-7, total n-9 and total saturated FA in the diets. FA discussed in the paper are marked in bold script and names are given in the column behind where appropriate.

**n-3 FA**		**n-6 FA**		n-7 FA	n-9 FA	saturated FA
C 18:3n3	LNA	C 18:2n6		C 16:1n7	C 16:1n9	C 14:0
C 18:4n3		C 18:3n6	LA	C 18:1n7	C 18:1n9	C 15:0
C 20:4n3		C 20:2n6		C 18:2n7	C 20:1n9	C 16:0
**C 20:5n3**	**EPA**	**C 20:4n6**	**AA**		C 22:1n9	C 17:0
C 22:5n3	DPA	C 22:4n6				C 18:0
**C 22:6n3**	**DHA**	C 22:5n6				C 20:0
						C21:0
						C 22:0
