# Supplementary material for: Fatty acid patterns of dog erythrocyte membranes after feeding of a fish-oil based DHA-rich supplement with a base diet low in n-3 fatty acids versus a diet containing added n-3 fatty acids
Source: Acta Vet Scand. 2011 Oct 24;53(1):57. doi: 10.1186/1751-0147-53-57 (PMC3213045; doi:10.1186/1751-0147-53-57)
Supplement: Additional file 2 — FA analysed in EM for CONT, ADD and FO (median and quartiles). [file 1751-0147-53-57-S2.PDF]

Median 2nd and 3rd quartiles of the proportions of n-7, n-9 and saturated (sat.) fatty acids (FA) in erythrocyte membranes (EM)
in control (CONT), additive (ADD) and fish oil (FO) group before and at week 1, 2, 4, 8, 12, 14 and 16 of experimental feeding.
Different superscript letters indicate statistically significant differences between different time points within the individual groups. ($p \leq 0.01$).
The FA included for the calculation of total n-3, n-6, n-7, n-9 and saturated FA are listed in additional file 1

		week	0	1	2	4	8	12	14	16
CONT	AA	25% quartile	28,45	28,875	28,25	28,2775	28,75	27,45		
		**median**	**29,35[a]**	**29,65[a]**	**29[a]**	**29,4[a]**	**29,65[a]**	**28,1[a]**		
		75% quartile	30,9	30,95	30,675	30,45	30,725	29,15		
	EPA	25% quartile	0,3475	0,32	0,3325	0,2925	0,2825	0,15		
		**median**	**0,395[a]**	**0,345[a]**	**0,425[a]**	**0,37[a]**	**0,325[a]**	**0,2[b]**		
		75% quartile	0,415	0,4425	0,485	0,4025	0,375	0,255		
	DHA	25% quartile	0,1725	0,205	0,185	0,1775	0,13	0,12		
		**median**	**0,225[a]**	**0,245[a]**	**0,2[a]**	**0,215[a]**	**0,14[b]**	**0,14[ab]**		
		75% quartile	0,3	0,315	0,2425	0,2475	0,1625	0,2425		
	total n-3 FA	25% quartile	1,3975	1,4275	1,4925	1,38	1,38	1,1375		
		**median**	**1,545[a]**	**1,58[a]**	**1,615[a]**	**1,475[a]**	**1,405[a]**	**1,265[b]**		
		75% quartile	1,6475	1,6725	1,7025	1,6625	1,635	1,355		
	total n-6 FA	25% quartile	44,425	44,475	43,95	43,2075	44,85	42,75		
		**median**	**44,7[ab]**	**44,9[ab]**	**44,25[a]**	**44,75[ab]**	**45,25[b]**	**43,15[c]**		
		75% quartile	44,85	45,425	44,725	45,2	45,325	43,4		
	total n-7 FA	25% quartile	3,045	2,89	2,8875	2,9175	2,915	2,88		
		**median**	**3,17[a]**	**3,11[a]**	**3,095[a]**	**3,09[a]**	**3,04[a]**	**3,04[a]**		
		75% quartile	3,2975	3,2375	3,1625	3,25	3,1725	3,165		
	total n-9 FA	25% quartile	10,575	10,575	10,65	10,6075	10,35	10,375		
		**median**	**10,7[a]**	**10,7[a]**	**10,85[a]**	**10,7[a]**	**10,55[a]**	**10,5[a]**		
		75% quartile	10,95	10,825	11,125	11,0375	10,725	10,775		
	total saturated FA	25% quartile	39,4375	39,0075	39,8975	38,9875	39,4775	41,6775		
		**median**	**39,91[a]**	**39,56[a]**	**40,25[a]**	**39,5[a]**	**39,665[a]**	**41,985[b]**		
		75% quartile	40,115	40,16	40,505	39,92	39,965	42,1875		

ADD

	week	0	1	2	4	8	12	14	16
	25% quartile	28,175	28,275	27,975	27,95	27,35	26,025	26,105	26,77
**AA**	**median**	**29,8**[a]	**29,7**[a]	**28,975**[ab]	**29**[abc]	**28**[bcd]	**26,6**[e]	**27,295**[de]	**27,45**[cde]
	75% quartile	30,65	30,625	29,85	29,425	28,625	26,875	27,765	28,2525
	25% quartile	0,3575	0,48	0,565	0,6625	0,6875	0,63	0,555	0,5025
**EPA**	**median**	**0,405**[a]	**0,52**[b]	**0,67**[bc]	**0,67**[c]	**0,76**[c]	**0,695**[c]	**0,61**[bc]	**0,565**[b]
	75% quartile	0,4525	0,63	0,7525	0,7475	0,9175	0,9	0,7775	0,625
	25% quartile	0,2075	0,6375	1,065	1,355	1,64	1,7125	1,3875	1,1625
**DHA**	**median**	**0,245**[a]	**0,76**[b]	**1,115**[c]	**1,52**[d]	**1,745**[ef]	**1,805**[f]	**1,61**[de]	**1,215**[c]
	75% quartile	0,2625	0,8225	1,185	1,5925	1,9925	1,94	1,73	1,38
	25% quartile	1,495	2,125	2,565	2,9175	3,19	3,2075	2,78	2,435
**total n-3 FA**	**median**	**1,585**[a]	**2,235**[b]	**2,705**[cd]	**3,025**[e]	**3,33**[f]	**3,3**[f]	**3,005**[de]	**2,6**[c]
	75% quartile	1,6225	2,315	2,86	3,2125	3,61	3,555	3,1075	2,82
	25% quartile	44,775	44	43,55	43,275	43,2	41,075	41,4675	42,1825
**total n-6 FA**	**median**	**45,05**[a]	**44,35**[ab]	**43,75**[c]	**43,95**[bc]	**43,6**[c]	**41,4**[d]	**42,16**[de]	**42,51**[e]
	75% quartile	45,3	45,125	44,125	44,475	43,775	41,875	42,3375	42,8025
	25% quartile	3,075	3,0125	2,9425	2,89	2,88	2,8475	3,2	3,05
**total n-7 FA**	**median**	**3,1**[ac]	**3,055**[abd]	**2,97**[ab]	**2,98**[abd]	**2,93**[b]	**2,96**[ab]	**3,35**[c]	**3,295**[cd]
	75% quartile	3,26	3,1675	3,0725	3,08	3,0175	3,11	3,8	3,4175
	25% quartile	10,4	10,375	10,35	10,25	10	10,275	10,3275	10,8075
**total n-9 FA**	**median**	**10,8**[a]	**10,6**[a]	**10,45**[a]	**10,55**[a]	**10,4**[a]	**10,45**[a]	**10,655**[a]	**11,27**[b]
	75% quartile	11,025	10,85	10,625	10,725	10,625	10,625	11,345	11,64
	25% quartile	38,9125	39,2575	39,485	38,8775	39,21	41,2	40,385	40,1
**total saturated FA**	**median**	**39,525**[a]	**39,625**[a]	**39,755**[a]	**39,555**[a]	**39,52**[a]	**41,755**[b]	**40,73**[c]	**40,37**[c]
	75% quartile	39,6575	39,795	40,08	40,05	40,2425	42,345	41,1025	40,9525

		week	0	1	2	4	8	12	14	16
FO	AA	25% quartile	28,9	29,3	28,675	28,325	28,65	27,075		
		**median**	**29,3[a]**	**29,7[a]**	**29,35[a]**	**28,85[a]**	**29,05[a]**	**27,55[b]**		
		75% quartile	30,025	30,65	29,925	29,9	29,25	28,1		
	EPA	25% quartile	0,3275	0,4625	0,705	0,875	0,99	0,845		
		**median**	**0,34[a]**	**0,55[b]**	**0,78[c]**	**0,965[d]**	**1,11[d]**	**0,985[d]**		
		75% quartile	0,3775	0,5975	0,8375	1,1025	1,2275	1,095		
	DHA	25% quartile	0,2375	0,485	0,6575	0,95	1,21	1,2675		
		**median**	**0,28[a]**	**0,53[b]**	**0,68[c]**	**1,025[d]**	**1,37[e]**	**1,39[e]**		
		75% quartile	0,31	0,5725	0,82	1,17	1,4925	1,51		
	total n-3 FA	25% quartile	1,3875	1,85	2,2925	2,7225	3,2325	3,1375		
		**median**	**1,485[a]**	**2,005[b]**	**2,38[c]**	**2,995[d]**	**3,39[e]**	**3,18[e]**		
		75% quartile	1,6075	2,0675	2,495	3,09	3,6725	3,435		
	total n-6 FA	25% quartile	44,45	44	43,2	42,85	42,525	40,925		
		**median**	**45,15[a]**	**44,25[b]**	**43,65[b]**	**43,1b[c]**	**42,85[c]**	**41,1[d]**		
		75% quartile	45,5	44,55	43,975	44,1	43,125	41,35		
	total n-7 FA	25% quartile	2,8975	3,0275	3,005	2,9475	3,0125	3,045		
		**median**	**3,065[a]**	**3,18[a]**	**3,16[a]**	**3,185[a]**	**3,15[a]**	**3,185[a]**		
		75% quartile	3,3825	3,37	3,33	3,385	3,3025	3,325		
	total n-9 FA	25% quartile	10,675	10,475	10,575	10,525	10,375	10,2		
		**median**	**10,75[a]**	**10,65[a]**	**10,7[a]**	**10,7[a]**	**10,6[a]**	**10,4[a]**		
		75% quartile	11,05	11	10,95	10,975	10,75	10,85		
	total saturated FA	25% quartile	39,045	39,3425	39,5975	38,95	39,465	41,8375		
		**median**	**39,425[a]**	**39,865[a]**	**39,84[a]**	**39,79[a]**	**39,955[a]**	**42,08[b]**		
		75% quartile	39,965	40,2275	40,135	40,265	40,4425	42,235		
