# Supplementary material for: Fatty acid patterns of dog erythrocyte membranes after feeding of a fish-oil based DHA-rich supplement with a base diet low in n-3 fatty acids versus a diet containing added n-3 fatty acids
Source: Acta Vet Scand. 2011 Oct 24;53(1):57. doi: 10.1186/1751-0147-53-57 (PMC3213045; doi:10.1186/1751-0147-53-57)
Supplement: Additional file 3 — Median 2nd and 3rd quartiles of the proportions of linoleic acid (LA), alpha linolenic acid (ALA) and docosapentaenoic acid (DPA) in erythrocyte membranes in control (CONT), additive (ADD) and fish oil (FO) group before and at week 1, 2, 4, 8, 12, 14 and 16 of experimental feeding. [file 1751-0147-53-57-S3.PDF]

Median 2nd and 3rd quartiles of the proportions of linoleic acid (LA), alpha linolenic acid (ALA) and docosapentaenoic acid (DPA) in erythrocyte membranes in control (CONT), additive (ADD) and fish oil (FO) group before and at week 1, 2, 4, 8, 12, 14 and 16 of experimental feeding.

Different superscript letters indicate statistically significant differences between different time points within the individual groups (all $p \leq 0.01$).

		Week	0	1	2	4	8	12	14	16
CONT	% LA	25% quartile	10.43	10.60	10.38	10.30	11.18	10.90		
		**Median**	**12.00^{a}**	**11.90^{a}**	**11.65^{a}**	**11.75^{a}**	**11.75^{a}**	**11.60^{a}**		
		75% quartile	12.43	12.48	12.35	12.68	12.38	12.28		
	% LNA	25% quartile	0.16	0.16	0.15	0.16	0.17	0.14		
		**Median**	**0.18^{a}**	**0.18^{a}**	**0.19^{a}**	**0.18^{a}**	**0.18^{a}**	**0.16^{a}**		
		75% quartile	0.20	0.21	0.20	0.20	0.21	0.18		
	% DPA	25% quartile	0.60	0.66	0.68	0.64	0.72	0.61		
		**Median**	**0.72^{a}**	**0.77^{a}**	**0.77^{a}**	**0.72^{a}**	**0.75^{a}**	**0.72^{a}**		
		75% quartile	0.78	0.84	0.87	0.86	0.82	0.79		
ADD	% LA	25% quartile	11.05	10.78	10.81	11.48	12.00	11.55	11.44	11.65
		**Median**	**11.80^{a}**	**11.20^{a}**	**11.15^{a}**	**11.70^{a}**	**12.15^{a}**	**11.80^{a}**	**11.99^{a}**	**11.93^{a}**
		75% quartile	12.68	12.18	11.88	12.30	12.70	12.75	12.61	12.44
	% LNA	25% quartile	0.17	0.16	0.17	0.16	0.17	0.15	0.12	0.14
		**Median**	**0.19^{a}**	**0.17^{a}**	**0.19^{a}**	**0.20^{a}**	**0.19^{a}**	**0.19^{a}**	**0.16^{a}**	**0.17^{a}**
		75% quartile	0.22	0.21	0.21	0.21	0.23	0.22	0.21	0.21
	% DPA	25% quartile	0.61	0.66	0.64	0.60	0.56	0.55	0.50	0.52
		**Median**	**0.67^{ab}**	**0.74^{b}**	**0.66^{bc}**	**0.65^{ab}**	**0.62^{ac}**	**0.59^{a}**	**0.57^{ac}**	**0.63^{ac}**
		75% quartile	0.76	0.77	0.77	0.67	0.69	0.63	0.69	0.65
FO	% LA	25% quartile	11.30	10.04	10.28	10.18	10.10	9.64		
		**Median**	**11.95^{a}**	**10.50^{a}**	**10.95^{a}**	**10.60^{a}**	**10.35^{a}**	**10.50^{a}**		
		75% quartile	12.58	11.43	11.40	11.60	11.05	11.13		
	% LNA	25% quartile	0.16	0.09	0.08	0.07	0.07	0.07		
		**Median**	**0.17^{a}**	**0.10^{b}**	**0.09^{bc}**	**0.08^{cd}**	**0.08^{d}**	**0.07^{d}**		
		75% quartile	0.21	0.12	0.09	0.09	0.08	0.08		
	% DPA	25% quartile	0.55	0.64	0.75	0.73	0.71	0.68		
		**Median**	**0.70^{a}**	**0.77^{a}**	**0.81^{a}**	**0.83^{a}**	**0.83^{a}**	**0.75^{a}**		
		75% quartile	0.77	0.89	0.87	0.88	0.90	0.87		
